# Successful Medical Management of Obstructive Mechanical Mitral Valve Thrombosis Using Low‐Dose, Ultraslow Infusion Thrombolytic Therapy: A Case Report

**DOI:** 10.1155/cric/7825905

**Published:** 2026-11-01

**Authors:** Kha Minh Nguyen, Huy Hoang Huynh, Loc Nguyen Minh Tran, Lan Thanh Phan, Sy Van Hoang

**Affiliations:** ^1^ Department of Internal Medicine School of Medicine University of Medicine and Pharmacy at Ho Chi Minh City Ho Chi Minh City Vietnam hcmut.edu.vn; ^2^ Department of Cardiology Cho Ray Hospital Ho Chi Minh City Vietnam choray.vn

**Keywords:** alteplase, case report, low-dose ultraslow thrombolysis, mechanical valve obstruction, prosthetic valve thrombosis

## Abstract

Obstructive mechanical valve thrombosis is a life‐threatening complication that requires emergent diagnosis and intervention. We report the case of a 61‐year‐old man who presented with dyspnea. He had undergone mechanical aortic and mitral valve replacement in 2010. Admission laboratory results revealed subtherapeutic anticoagulation with an international normalized ratio of 1.09. Transthoracic and transesophageal echocardiography confirmed obstructive mechanical mitral valve thrombosis, with a 17 × 25 mm mass showing imaging features consistent with subacute thrombus and suggestive of concomitant pannus, which restricted the motion of one leaflet and increased the mean transmitral pressure gradient to 23 mmHg. Because of the patient′s substantial surgical risk and the family′s refusal of surgical intervention, the patient received low‐dose, ultraslow thrombolytic therapy with alteplase administered as a continuous intravenous infusion of 25 mg over 25 h. The patient tolerated the regimen well, with no hemorrhagic or systemic embolic complications. Postintervention echocardiographic evaluation showed hemodynamic recovery (mean pressure gradient decreased to 3 mmHg). The patient was discharged on vitamin K antagonist therapy with planned close international normalized ratio monitoring. This case demonstrates that low‐dose, ultraslow alteplase infusion may be a feasible treatment option for carefully selected patients with obstructive mechanical prosthetic valve thrombosis when surgery is considered high risk or declined.

Learning Objective

This case demonstrates the successful management of obstructive prosthetic mitral valve thrombosis with low‐dose, ultraslow alteplase in a high‐risk patient. Differentiating thrombus from pannus by echocardiography is crucial, because thrombotic obstruction is reversible with fibrinolysis, whereas pannus does not respond to fibrinolysis and may persist. Prevention of recurrent thrombosis requires strict long‐term anticoagulation and regular international normalized ratio monitoring.

## 1. Introduction

Mechanical prosthetic valve thrombosis is an uncommon but potentially fatal complication after mechanical heart valve replacement. Although mechanical prostheses are durable, they remain thrombogenic and require lifelong vitamin K antagonist therapy. The annual incidence of prosthetic valve thrombosis ranges from 0.1% to 5.7%, with higher risk in the mitral or tricuspid position, older generation prostheses, atrial fibrillation, ventricular dysfunction, and subtherapeutic anticoagulation [1, 2]. Obstructive prosthetic valve thrombosis is particularly dangerous because restricted leaflet motion rapidly increases transprosthetic gradients, leading to acute pulmonary edema, decompensated heart failure, pulmonary hypertension, or cardiogenic shock. Clinicians should suspect this diagnosis when a patient with a mechanical valve develops new dyspnea, muffled prosthetic clicks, a new murmur, or elevated gradients, especially with a subtherapeutic international normalized ratio [1].

Management of symptomatic left‐sided obstructive mechanical prosthetic valve thrombosis remains challenging because both surgery and fibrinolysis carry risks. Earlier thrombolytic experience showed efficacy but also limitations. In a single‐center series of 127 episodes, fibrinolysis achieved complete success in 70.9% of episodes, with lower success in mitral than aortic prostheses [3]. Current recommendations support individualized heart team decision‐making. The 2025 European Society of Cardiology/European Association for Cardio‐Thoracic Surgery guideline recommends urgent or emergency valve replacement for critically ill patients without serious comorbidity while recognizing low‐dose slow‐infusion fibrinolysis as an option after heart team assessment [4]. The 2020 American College of Cardiology/American Heart Association guideline similarly recommends urgent treatment with either slow‐infusion, low‐dose fibrinolysis or emergency surgery for symptomatic left‐sided mechanical prosthetic valve thrombosis [5]. Data from the TROIA trial, PROMETEE trial, and HATTUSHA study support low‐dose slow or ultraslow alteplase infusion in selected patients, especially when surgery is high risk or declined [6–8]. We report the case of a high‐risk patient with obstructive mechanical mitral valve thrombosis caused by a large subacute thrombus with echocardiographic features suggestive of concomitant pannus, successfully treated with low‐dose, ultraslow alteplase infusion. This case report was prepared in accordance with the CARE guidelines (File S1).

## 2. Case Presentation

A 61‐year‐old man presented to the Emergency Department of Cho Ray Hospital with progressive dyspnea for 10 days. He had worsening shortness of breath on mild exertion without chest pain. His medical history included mechanical aortic and mitral valve replacement with tricuspid annuloplasty in 2010. He had been maintained on long‐term anticoagulation with acenocoumarol, although adherence had been inconsistent. The postoperative course was uneventful, and no prosthetic valve dysfunction had been documented before the current admission. His other medical conditions included heart failure, ischemic heart disease, atrial fibrillation, hypertension, Stage 3 chronic kidney disease, and dyslipidemia. There was no relevant family history of valvular heart disease or thrombotic disorders, and no major psychosocial factors affecting treatment or follow‐up were identified.

On admission, he was awake but agitated. Blood pressure was 110/70 mmHg, heart rate was 92 beats/min, respiratory rate was 26 breaths/min with accessory muscle use, and oxygen saturation was 97% on 3 L/min of oxygen via nasal cannula. Physical examination revealed jugular venous distention, bilateral crackles, an irregularly irregular rhythm, and muffled mechanical mitral valve clicks. Initial laboratory testing showed markedly subtherapeutic anticoagulation with an international normalized ratio of 1.09. Serum creatinine was 1.66 mg/dL, estimated glomerular filtration rate was 46.61 mL/min/1.73 m^2^, high‐sensitivity troponin T was 32.04 pg/mL, and N‐terminal pro‐B‐type natriuretic peptide was 1945.2 pg/mL.

Emergency transthoracic echocardiography showed a mildly reduced left ventricular ejection fraction of 43%. The mechanical aortic valve was well seated, with normal function and a mean pressure gradient of 4.6 mmHg. In contrast, the mechanical mitral valve showed restricted motion of one leaflet, a peak velocity of 2.8 m/s, and a mean transmitral gradient of 19.4 mmHg. The estimated pulmonary artery systolic pressure was markedly elevated at 70 mmHg, indicating clinically significant mechanical mitral valve obstruction. Transesophageal echocardiography confirmed restricted motion of one mitral prosthetic leaflet (Video S1), with a peak velocity of 3.09 m/s and a mean pressure gradient of 23 mmHg (Figure 1A), consistent with severe prosthetic mitral obstruction. It also demonstrated a 17 × 25 mm mass on the mechanical mitral valve apparatus, with imaging features consistent with a subacute thrombus (Figure 1B). Multiple dense echogenic annular structures and thickening of the valve apparatus to 5–6 mm were suggestive of concomitant pannus. The principal diagnostic challenge was determining the relative contribution of thrombus and pannus because both can restrict prosthetic leaflet motion and may coexist. The acute symptom progression, markedly subtherapeutic international normalized ratio, spontaneous echocardiographic contrast, and a large prosthetic valve mass favored thrombus as the dominant reversible component, whereas the dense annular structures and valve thickening were suggestive of an associated pannus component (Table 1). Biatrial enlargement was also present.

**Figure 1:**
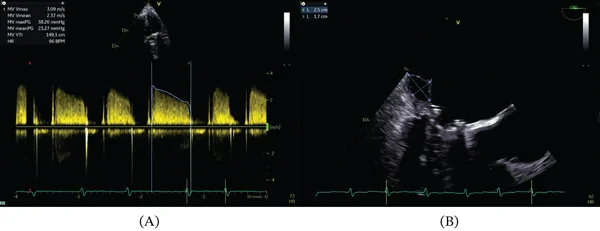
Transesophageal echocardiography before thrombolytic therapy demonstrating obstructive mechanical mitral valve thrombosis. (A) Continuous‐wave Doppler showing severe prosthetic mitral obstruction, with a peak velocity of 3.09 m/s and a mean pressure gradient of 23.27 mmHg. (B) Two‐dimensional imaging showing a 17 × 25 mm echogenic mass on the mechanical mitral valve apparatus, with additional imaging features suggestive of concomitant pannus.

**Table 1:** Clinical and echocardiographic features differentiating thrombus from pannus [9, 10].

Feature	Favors thrombus	Favors pannus	Findings in this case
Chronology	May occur at any time; late thrombosis may coexist with pannus	Usually occurs > 12 months after surgery, often several years later	Mechanical valve replacement in 2010; symptoms developed over approximately 10 days
Relation to anticoagulation	Strongly associated with inadequate anticoagulation	Weak relationship with anticoagulation status	International normalized ratio of 1.09 with inconsistent anticoagulant adherence
Location	More commonly involves the mitral prosthesis; may be supra‐ or subvalvular	More commonly associated with the aortic prosthesis and periannular/subvalvular tissue	Mass involving the mechanical mitral valve apparatus with periannular thickening
Morphology	Usually larger and irregular; may be attached to the leaflet or hinge region	Usually smaller, fixed tissue involving the sewing ring	Large 17 × 25 mm mass with additional dense periannular tissue
Echogenicity	Relatively low or soft echodensity	Dense, highly echogenic tissue, often similar to the prosthetic ring	Mixed echogenic appearance with dense periannular structures
Leaflet/disc motion	Restricted motion is common	Restriction may occur but is not always present	One mechanical mitral leaflet was markedly restricted
Response to fibrinolysis	Reduction of the mass and restoration of valve function support a thrombotic component	Fibrous pannus is not expected to resolve with fibrinolysis	Leaflet motion improved and transmitral gradient markedly decreased; a small residual mass and periannular tissue remained
Overall assessment	—	—	Thrombus strongly supported; concomitant pannus suspected on imaging but not definitively confirmed

## 3. Treatment, Outcome, and Follow‐Up

After confirmation of obstructive mechanical mitral valve thrombosis, a multidisciplinary heart team discussion was held. Because of heart failure, atrial fibrillation, chronic kidney disease, severe pulmonary hypertension, previous multiple‐valve surgery, and overall comorbidity burden, surgical reintervention was considered to carry substantial operative risk. The patient′s family also declined surgery. Systemic thrombolytic therapy was therefore selected.

Intravenous unfractionated heparin, initiated on admission, was discontinued before fibrinolysis. Alteplase 25 mg was infused continuously over 25 h at 1 mg/h, without a bolus, using a low‐dose, ultraslow protocol adapted from the PROMETEE trial [7]. During treatment, the patient was closely monitored for neurological deterioration, hemodynamic instability, bleeding, and systemic embolism. The patient reported marked improvement in dyspnea after completion of the thrombolytic infusion. No hemorrhagic or systemic embolic complications were observed. Immediate transthoracic echocardiographic reassessment demonstrated marked hemodynamic recovery: Mean pressure gradient decreased from 23 to 3 mmHg, peak velocity decreased to 1.28 m/s, and effective orifice area improved to 2.04 cm^2^.

Posttreatment transesophageal echocardiography showed a mildly reduced left ventricular ejection fraction of 43%, left ventricular dilation and hypertrophy, generalized four‐chamber enlargement, moderate tricuspid regurgitation, and a lower estimated pulmonary artery systolic pressure of 49 mmHg. The mechanical mitral valve was well seated, with restored leaflet motion after thrombolysis (Video S2). Doppler assessment of the mitral prosthesis demonstrated substantial hemodynamic improvement, with a peak velocity of 1.73 m/s and a mean gradient of 4.67 mmHg (Figure 2A), indicating relief of the previous obstructive physiology. A small residual thrombus measuring 4 × 8 mm was observed between the mitral leaflet hinges without restricting leaflet motion, and suspected pannus involving the annulus and leaflets persisted (Figures 2B–D). In addition, a large left atrial thrombus measuring 28 × 38 × 20 mm was detected along the posterior and lateral atrial walls near the coronary sinus, extending toward the mechanical mitral valve hinge (Figure 2E). The mechanical aortic valve was well seated, with normal leaflet mobility, acceptable Doppler parameters (Figure 2F), and suspected annular pannus.

**Figure 2:**
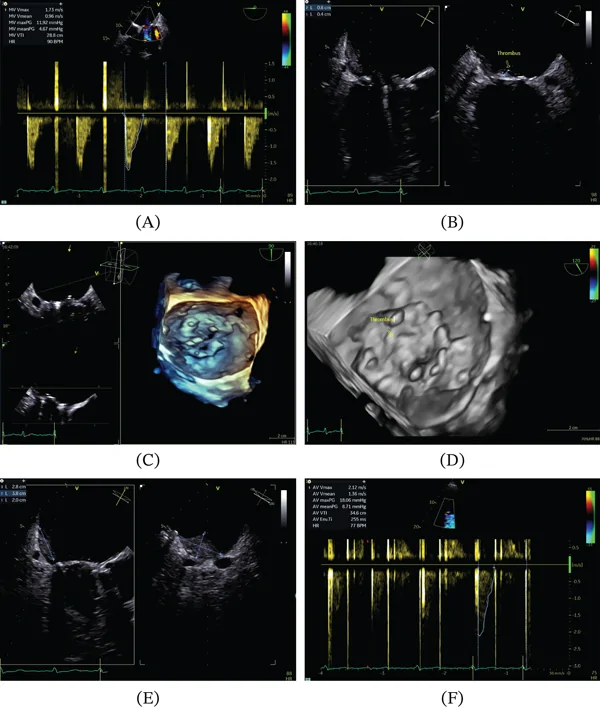
Posttreatment transesophageal echocardiography after completion of low‐dose, ultraslow alteplase infusion. (A) Doppler assessment of the mechanical mitral prosthesis showing substantial hemodynamic improvement, with a peak velocity of 1.73 m/s and a mean pressure gradient of 4.67 mmHg, indicating relief of the previous obstructive physiology. (B) Biplane imaging showing a small nonobstructive thrombus (arrow) measuring 4 × 8 mm between the leaflet hinges. (C) Three‐dimensional rendering of the mechanical mitral prosthesis demonstrating a well‐seated valve with residual echogenic tissue suggestive of pannus. (D) Magnified three‐dimensional rendering showing the small residual thrombus (arrow) at the leaflet hinge region. (E) Biplane imaging showing a large left atrial thrombus measuring 28 × 38 × 20 mm along the posterior and lateral atrial walls near the coronary sinus, extending toward the mechanical mitral valve hinge. (F) Doppler assessment of the mechanical aortic prosthesis showing acceptable gradients.

Unfractionated heparin was then restarted, with bridging to acenocoumarol. A subsequent predischarge transthoracic echocardiogram demonstrated sustained improvement in mechanical mitral valve hemodynamics, with a peak velocity of approximately 1.8 m/s, a mean transmitral gradient of 5 mmHg, and a mitral velocity time integral of 52.5 cm. This transthoracic echocardiogram did not specifically reassess the residual prosthetic thrombus or the left atrial thrombus, and no repeat transesophageal echocardiography was performed before discharge. After clinical stabilization and resolution of dyspnea, the patient was discharged with a scheduled follow‐up plan emphasizing strict outpatient international normalized ratio monitoring and anticoagulation optimization.

During subsequent outpatient follow‐up, the patient remained clinically stable. No further echocardiography was performed after discharge. Anticoagulation control remained suboptimal owing to poor medication adherence, prompting counseling of both the patient and family regarding consistent anticoagulant use and regular international normalized ratio monitoring. No recurrent prosthetic valve obstruction or thromboembolic event was documented in the available records.

## 4. Patient Perspective

At the time of discharge, the patient reported complete resolution of dyspnea. He and his family were counseled regarding medication use, adherence to anticoagulation therapy, regular international normalized ratio monitoring, and the importance of scheduled follow‐up.

## 5. Discussion

This case highlights three clinically important issues in mechanical prosthetic valve obstruction. First, differentiating thrombus from pannus is essential because thrombus may respond to fibrinolysis, whereas pannus is fibrotic tissue and generally requires surgery if it causes significant obstruction [1, 9]. In this patient, the very low international normalized ratio, acute symptom progression, spontaneous echocardiographic contrast, and a large mass attached to the prosthetic mitral apparatus strongly supported thrombotic obstruction as the main reversible component (Table 1). However, the annular echogenic structures and valve thickening were suggestive of concomitant pannus, reflecting the complexity of late prosthetic valve obstruction.

Second, transesophageal echocardiography was central to diagnosis and therapeutic planning. Prosthetic valve assessment should integrate leaflet mobility, Doppler gradients, mass location, morphology, and echogenicity [9]. Pannus is usually dense, annular, and confined near the sewing ring, whereas thrombus is often larger, less echogenic, more irregular, and strongly associated with inadequate anticoagulation. In this case, transthoracic echocardiography detected obstructive physiology, whereas transesophageal echocardiography clarified the mechanism by demonstrating a large thrombus with accompanying imaging features suggestive of pannus.

Third, thrombus burden remains an important consideration when fibrinolysis is contemplated. Although low‐dose slow or ultraslow fibrinolytic regimens have achieved favorable outcomes in selected patients with mechanical prosthetic valve thrombosis, increasing thrombus size has been associated with less favorable treatment outcomes. In the TROIA trial, a thrombus area > 0.9 cm^2^ was associated with increased embolic events [6], whereas the PROMETEE trial found greater thrombus area to be associated with thrombolytic complications and unsuccessful treatment [7]. The HATTUSHA study nevertheless demonstrated that thrombolysis could be successful in patients with substantial thrombus burden, with a median thrombus area of 1.1 cm^2^ (interquartile range, 0.9–1.3 cm^2^) and an overall treatment success rate of 90.4% [8]. Consistent with these concerns, current American College of Cardiology/American Heart Association guidance lists a large clot (> 0.8 cm^2^) among the factors favoring surgery over fibrinolysis [5]. Direct quantitative comparison with the present case is limited because these studies primarily quantified thrombus burden by planimetric area, whereas the thrombus in our patient was reported by linear dimensions (17 × 25 mm). However, its substantial size still indicated a considerable thrombus burden and remained an important factor in treatment selection.

In this patient, the decision to pursue low‐dose, ultraslow alteplase was supported by a combination of favorable features, including hemodynamic stability, markedly subtherapeutic anticoagulation, a first documented episode of prosthetic valve thrombosis, imaging findings favoring thrombus as the predominant reversible component, and preserved function of the mechanical aortic prosthesis without evidence of obstruction. In parallel, the substantial operative risk associated with redo surgery and the family′s decision to decline surgical intervention further shifted the balance toward fibrinolysis. A large left atrial thrombus was not identified before fibrinolysis; had it been detected, it would have represented an additional factor favoring surgery over fibrinolysis [5], although the patient′s operative risk would still have weighed against surgical reintervention. Taken together, these considerations supported an individualized fibrinolytic strategy. The subsequent marked reduction in transmitral gradient, symptomatic improvement, and absence of major complications were consistent with a favorable treatment response.

Despite the favorable outcome, the findings of this case should not be generalized to all patients with left‐sided obstructive prosthetic valve thrombosis. Careful patient selection, exclusion of contraindications, intensive monitoring, and close posttreatment anticoagulation control remain essential. For long‐term management, the markedly subtherapeutic admission international normalized ratio of 1.09 represented an important modifiable risk factor, underscoring the need for patient education, consistent vitamin K antagonist therapy, and regular international normalized ratio surveillance to reduce the risk of recurrence.

## 6. Strengths and Limitations

Our case demonstrates the value of comprehensive echocardiographic assessment to characterize the mechanism and severity of prosthetic valve obstruction, together with serial imaging to assess the hemodynamic response to treatment. It also describes the use of low‐dose, ultraslow alteplase in a difficult‐to‐treat patient with a substantial prosthetic thrombus burden, with no major bleeding or clinical thromboembolism observed. However, cardiac computed tomography and histopathologic confirmation were unavailable, limiting definitive characterization of the residual echogenic tissue and differentiation between thrombus and pannus. Although predischarge echocardiography documented sustained hemodynamic improvement, no further echocardiography or cardiac computed tomography was performed after discharge to characterize the evolution of the residual prosthetic thrombus and the large left atrial thrombus. Because the left atrial thrombus was first identified on posttreatment transesophageal echocardiography, it could not be determined whether it had been present but unrecognized before fibrinolysis or developed during the interval. Finally, because this is a single case, these findings should not be generalized to routine fibrinolysis for patients with large left‐sided prosthetic thrombi.

## 7. Conclusion

Obstructive mechanical mitral valve thrombosis is a cardiovascular emergency requiring rapid recognition, multimodality imaging, and individualized heart team management. This case demonstrates successful treatment of a large subacute thrombus with suspected concomitant pannus using low‐dose, ultraslow alteplase infusion in a patient with substantial surgical risk whose family declined surgery. The regimen achieved rapid echocardiographic and hemodynamic recovery without hemorrhagic or systemic embolic complications. In selected high‐risk patients, low‐dose, ultraslow fibrinolysis may be an effective alternative to surgical reintervention, provided that careful monitoring and long‐term anticoagulation optimization are ensured.

## Funding

No funding was received for this manuscript.

## Consent

Written informed consent was obtained from the patient for publication. Institutional ethical approval was not required for this single case report.

## Conflicts of Interest

The authors declare no conflicts of interest.

## Supporting Information

Additional supporting information can be found online in the Supporting Information section.

## Supporting information


**Supporting Information 1** File S1: CARE checklist for this case report.

**Supporting Information 2** Video S1: Transesophageal echocardiography before thrombolytic therapy showing restricted motion of one mechanical mitral valve leaflet.

**Supporting Information 3** Video S2: Transesophageal echocardiography after thrombolytic therapy showing restored mechanical mitral valve leaflet motion.

## Data Availability

The data that support the findings of this study are available on request from the corresponding author. The data are not publicly available due to privacy or ethical restrictions.
